# A Cognitive Perspective on Information Frictions in Labor Markets

**DOI:** 10.3390/e27121182

**Published:** 2025-11-21

**Authors:** Zeqiang Zhang, Ruxin Chen

**Affiliations:** 1 Institute of Neural Information Processing, Ulm University, 89075 Ulm, Germany; zeqiang.zhang@uni-ulm.de; 2 Department of Economics, Nagoya University, Nagoya 464-8601, Aichi, Japan

**Keywords:** Free Energy Principle, active inference, information frictions, labor market matching, Shannon entropy, macroeconomic dynamics, search-and-matching framework

## Abstract

During the Great Recession, labor markets often exhibit a slow unemployment recovery and persistent outward shifts in the Beveridge curve, which suggests a decline in the efficiency of the job-matching process. While it is often explained by worker search intensity, we argue that the direction of search behavior also matters by proposing a stylized theoretical model based on the Free Energy Principle. Through modeling agents who actively divide their effort between applying for jobs and learning about the market’s new state, our framework shows that agents endogenously shift effort from applications to learning when their uncertainty is high. Building on this micro-foundation, we design a macroeconomic model where matching efficiency is no longer an external parameter but is instead governed by two cognitive factors: the share of unemployed workers with misaligned beliefs and the average learning effort of the informed. Simulation results show that a structural shock will divert effort to learning and depress matching by creating widespread uncertainty, and the subsequent slow recovery is governed by the realignment of collective beliefs. Our work provides a cognitive explanation for this observed persistence of unemployment and the shift of the Beveridge curve.

## 1. Introduction

Information frictions are a cornerstone of modern labor economics, giving rise to unemployment and inefficiencies in the matching process between workers and firms. In the canonical Diamond–Mortensen–Pissarides (DMP) framework, the job-finding rate depends on unemployment and vacancies via an aggregate matching function [1,2]. A common limitation is that the function’s efficiency is treated as exogenous or hit by ad hoc shocks, offering limited micro-foundations for how agents collect, process, and act on information, and how their cognitive states propagate to aggregate outcomes.

This limitation is empirically significant. A recurring fact in labor economics is that severe downturns may cause large and persistent outward shifts of the Beveridge curve, indicating a decline in matching efficiency, as shown during the Great Recession [3]. To measure matching efficiency, economists have traditionally focused on worker search intensity, such as the time spent searching or the number of applications sent [1,4]. However, empirical evidence suggests that the effectiveness of that search is also important, as changes in the composition of the unemployed pool can mechanically reduce the aggregate matching rate, even if individual search effort is high [5,6]. This ineffectiveness is often the result of mismatch unemployment [7], where the attributes of job seekers do not align with the demands of available jobs. This suggests the central problem may not only be a simple lack of effort, but also a misdirection of that effort.

This misdirection is related to a direct consequence of the Knightian uncertainty that arises after major structural shocks. Economic theory distinguishes between risk, where agents know the probabilities of future events, and Knightian uncertainty, where these probabilities are unknown, unreliable, or not even formulable [8]. The trends in modern economies, such as technological automation or pandemics, are not simple risks. They are structural shocks that generate Knightian uncertainty, instantly making agents’ old models of the economy inaccurate.

In reality, a significant component of a job seeker’s problem is this Knightian uncertainty: not merely finding offers, but first understanding the nature of the market itself. Which skills are in demand? Which industries are growing? Faced with this unknown, an agent cannot simply optimize before learn the new state of the world. In contrast to standard models that assume known distributions, we take an explicitly information-theoretic view: the uncertainty in beliefs can be quantified by Shannon entropy, and the dispersion of beliefs across workers is a sufficient statistic for the complexity of the information environment.

Specifically, we develop a stylized theoretical model as a foundational cognitive framework based on the Free Energy Principle (FEP) from theoretical neuroscience and biology [9]. Our choice of the FEP is methodologically motivated. While other frameworks (e.g., expected utility theory [10] or reinforcement learning [11]) could be engineered to model this trade-off, they would likely require ad hoc assumptions about the cost of uncertainty or the reward for learning. The FEP provides a more parsimonious and unifying alternative. It posits that any self-organizing system, including a human agent, acts to minimize a quantity known as “variational free energy”. This minimization process implicitly reduces the long-term average of “surprise”—the improbability of sensory states. Under the active inference formulation of this principle, agents take actions to minimize expected free energy. This objective naturally decomposes into two parts: pursuing preferred outcomes (pragmatic value) and resolving uncertainty about the environment (epistemic value).

We apply this framework to a job seeker who must allocate their finite effort between two activities: (1) information gathering to reduce uncertainty about the current state of the labor market, and (2) direct job search activities (e.g., submitting applications) to secure employment. This provides a formal basis for the intuition that agents in uncertain environments should prioritize learning.

Our analysis proceeds in two stages. We first use a toy microeconomic model of an individual worker to demonstrate how agents optimally balance belief updating (learning) with job seeking (applying) under the FEP. Following a structural shock, agents temporarily reallocate effort from applications to learning until their internal models realign. We then extend this individual behavior to the macro market by aggregating a continuum of such agents, deriving a macro model where two cognitive state variables endogenously govern matching efficiency. We then demonstrate qualitatively that this cognitive channel generates rich dynamics that match established real-world economic phenomena. Specifically, it provides a cognitive mechanism that can help account for the persistent shifts in the Beveridge curve observed after major recessions, offering a theoretical proof-of-concept for how collective learning dynamics can endogenously drive a persistence of unemployment.

This paper thus addresses three core questions: (1) How can a job seeker deal with the trade-off between job applications and information gathering? (2) How do these micro-level cognitive processes aggregate to shape the macroeconomic matching function? (3) How does this endogenous cognitive channel influence the labor market’s dynamic response to structural shocks?

The paper is structured as follows. Section 2 introduces the theoretical framework of the Free Energy Principle and Active Inference. Section 3 presents the simplified microeconomic model of the individual job seeker and simulation results. Section 4 develops the aggregate macroeconomic model. Section 5 analyzes the model’s dynamics and discusses its implications. Section 6 concludes the contribution and future work.

## 2. Theoretical Framework: The Free Energy Principle

To endogenize the process of information acquisition and belief formation in labor markets, we employ the FEP as a normative framework for decision making under uncertainty. The FEP posits a single objective function that governs both belief updating and the selection of actions, providing a unified model of behavior for a rational agent.

### 2.1. Belief Updating via Free Energy Minimization

We model an agent who must infer the unobserved state of the world, θ (e.g., the state of the labor market), from sensory observations, *s* (e.g., job postings, interview outcomes). The agent is equipped with a generative model, a joint probability distribution p(s,θ), which represents their internal hypotheses about how hidden states generate observations.

The fundamental objective of the agent is to minimize long-term “surprise”, defined as the negative log-probability for an outcome in information theory:

(1) Surprise(s)=−logp(s).

Direct minimization of surprise is intractable, as it requires knowing the marginal probability of the observation

(2) p(s)=∫p(s,θ)dθ.

Instead, the agent minimizes an upper bound on surprise known as variational free energy, *F* (see Appendix A.1). For a given observation *s* and an approximate posterior belief q(θ), free energy is defined as:

(3) $$F\left(s,q\right)=E_{q(\theta )}\left[\log q\left(\theta \right)-\log p\left(s,\theta \right)\right].$$

Minimizing this functional with respect to the agent’s beliefs q(θ) is equivalent to making those beliefs a better approximation of the true posterior p(θ|s), thereby rendering observations less surprising. The free energy functional can be rearranged into a more intuitive form that highlights the trade-off inherent in belief formation (see Appendix A.2):

(4) $$F=\underset{\mathrm{Complexity}}{\underset{︸}{D_{KL}\left[q(\theta )||p(\theta )\right]}}-\underset{\mathrm{Accuracy}}{\underset{︸}{E_{q(\theta )}\left[\log p(s|\theta )\right]}}.$$

Here, “Accuracy” is the expected log-likelihood of the observations given the agent’s beliefs, compelling the model to fit the data. “Complexity” is the Kullback–Leibler (KL) divergence between the posterior belief q(θ) and the prior belief p(θ). This term penalizes beliefs that deviate excessively from prior knowledge, acting as a form of Occam’s razor that regularizes the solution and prevents overfitting to noisy data. The minimization of free energy thus furnishes a Bayes-optimal process of belief updating.

### 2.2. Action Selection via Expected Free Energy Minimization

The FEP extends naturally to action selection through the framework of Active Inference, which posits that agents choose actions that minimize the expected free energy of future outcomes. The expected free energy, denoted by *G*, quantifies the anticipated divergence between an agent’s beliefs and the generative model of its environment under a given policy or action $a_{t}$. Formally, the expected free energy of action $a_{t}$ is given by:

(5) $$G\left(a_{t}\right)=E_{q(o_{t+1},s_{t+1}|a_{t})}\left[\log q\left(s_{t+1}|o_{t+1}\right)-\log p\left(o_{t+1},s_{t+1}\right)\right],$$

where $o_{t+1}$ and $s_{t+1}$ denote the predicted future observations and latent states, respectively. This expression can be decomposed into two interpretable components that correspond to distinct economic motivations, pragmatic value and epistemic value (see Appendix A.3):

(6) $$G\left(a_{t}\right)=\underset{\mathrm{Pragmatic}\;\mathrm{Value}}{\underset{︸}{E_{q(o_{t+1}|a_{t})}\left[-\log p\left(o_{t+1}\right)\right]}}+\underset{\mathrm{Epistemic}\;\mathrm{Value}}{\underset{︸}{E_{q(o_{t+1},s_{t+1}|a_{t})}\left[\log q\left(s_{t+1}|o_{t+1}\right)-\log q\left(s_{t+1}|a_{t}\right)\right]}}$$

The first term, referred to as pragmatic value (or expected utility), captures the expected divergence between predicted observations and the agent’s prior preferences, encoded as the distribution $p(o_{t+1})$. Minimizing this term leads to actions that are likely to generate outcomes aligning with the agent’s goals—for instance, obtaining a job offer in the labor market setting. Therefore, this component of *G* is formally analogous to the classical notion of maximizing expected utility.

The second term, known as epistemic value (or information gain), measures the expected reduction in uncertainty about latent states resulting from new observations. It is expressed as the anticipated KL divergence between the posterior belief after observing $o_{t+1}$, given by $q(s_{t+1}|o_{t+1})$, and the prior predictive belief before the observation, $q(s_{t+1}|a_{t})$. This term reflects the intrinsic value of acquiring information and thus promotes exploratory actions that enhance the agent’s understanding of its environment.

In the context of our labor market model, a job seeker selects actions to allocate effort between job applications and information gathering with the goal of minimizing expected free energy. This framework provides a formal account of the trade-off between exploitation and exploration. By integrating both pragmatic and epistemic considerations into a unified decision-making criterion, the expected free energy formulation offers a theoretically grounded explanation for adaptive labor market behavior under uncertainty.

## 3. A Microeconomic Model of Job Search

We now apply the active inference framework to model the decision-making process of an individual unemployed worker. In this simplified micro model, the agent’s core economic problem is to optimally allocate a finite budget of effort between two distinct activities: direct job applications, which serve the pragmatic goal of securing employment, and information gathering, which serves the epistemic goal of reducing uncertainty about the true state of the labor market.

### 3.1. The Worker’s Problem

Consider a worker navigating a labor market that may exist in one of two latent states, denoted by S∈{A,B}. State A represents an “old economy” in which traditional skills are in demand, whereas State B corresponds to a “new economy” that requires a different, more modern skill set. The worker does not directly observe the true state of the market but instead forms a belief over these two possibilities, represented by the probability distribution [p(S=A),p(S=B)].

Each period, the worker is endowed with a fixed amount of effort, normalized to one unit. This effort must be allocated between two competing activities: information gathering and job application. Let α∈[0,1] denote the fraction of effort devoted to acquiring information, which may include activities such as reading industry reports, attending networking events, and researching emerging trends in skill demand. The remaining fraction, 1−α, is used to directly search and apply for jobs.

The success of the worker’s job application depends on two factors: the intensity of application effort and the accuracy of their beliefs about the current market state. A mismatch between the worker’s belief-driven strategy and the actual state of the market leads to a reduced probability of obtaining a good job, such as focusing on new-economy roles when the economy is in fact in the old-economy state.

Within the active inference framework, the allocation of effort (α) between exploration and exploitation is driven by a trade-off between the value of reducing uncertainty (epistemic value) and the immediate value of securing employment (pragmatic value). When the worker’s belief about the state of the labor market is highly uncertain, characterized by high entropy in the belief distribution, the epistemic value of acquiring more accurate information is substantial. In this case, the worker is incentivized to choose a higher α in order to prioritize learning. Conversely, when the worker’s belief is relatively certain and entropy is low, the epistemic drive diminishes. Under such conditions, the worker is more likely to allocate their effort toward the pragmatic objective of job acquisition, choosing a lower value of α in favor of maximizing direct applications.

### 3.2. Simulation Results

To demonstrate the behavioral implications of our model, we conduct a numerical simulation of a single agent’s decision-making process over time. The objective is to illustrate the core prediction derived from the active inference framework: that an agent’s allocation of effort towards information gathering is endogenously and dynamically determined by their level of subjective uncertainty.

We simulate a scenario involving an unanticipated structural break in the labor market. The environment begins in a stable “old market” (State A), a state which the agent correctly perceives with high confidence. At time t=30, the market undergoes a permanent and unexpected transition to a “new market” (State B). The agent must infer this change from ongoing observations. The details of the simulation can be found in Appendix B.

**Figure 1:**
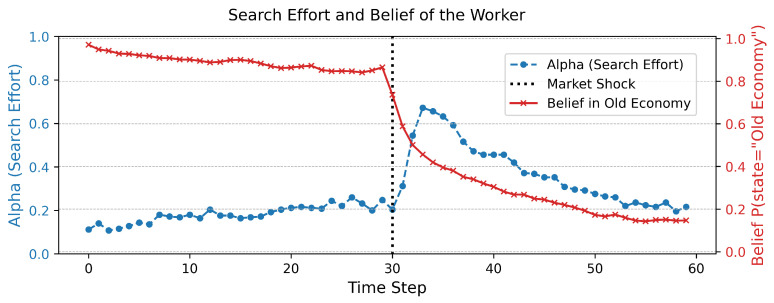
Dynamic response of beliefs and search effort to a structural shock. The figure plots the agent’s belief in the initial market state (State A) and the proportion of effort allocated to information gathering (α). A permanent structural shock to State B occurs at t=30.

Figure 1 presents the time-series evolution of the agent’s belief in the original market state (State A) and their corresponding allocation of effort to information gathering (α). Prior to the structural break (t<30), the agent is confident in their correct model of the world, and consequently, the effort allocated to information gathering remains at a minimal baseline level. The pragmatic goal of job application is dominant. Immediately following the shock at t=30, the agent’s model begins to generate significant prediction errors, which drives a rapid belief revision process. As the agent’s certainty about State A collapses, their allocation of effort to information gathering, α, increases sharply. This period of intense epistemic activity persists until the agent’s beliefs converge towards the new market reality (State B). Once a new, confident model is established, uncertainty is resolved, and α recedes to its low baseline, allowing effort to be reallocated to pragmatic job applications.

**Figure 2:**
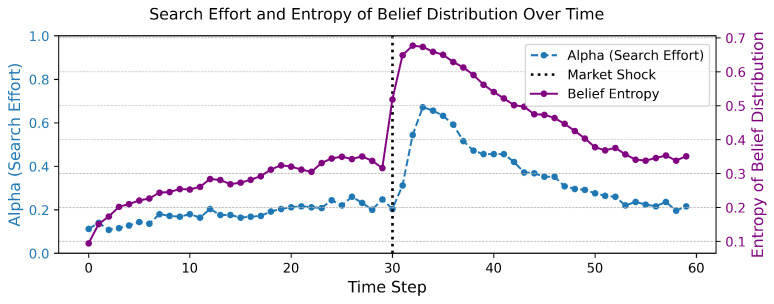
The evolution of subjective uncertainty following a structural shock. The figure shows the Shannon entropy of the agent’s belief distribution over time. Entropy serves as a formal measure of the agent’s subjective uncertainty, which peaks during the period of belief revision.

The underlying driver of this dynamic allocation of effort is the agent’s subjective uncertainty, which can be quantified by the Shannon entropy of their belief distribution. Figure 2 plots this belief entropy over time. The trajectory of entropy precisely mirrors the agent’s information-gathering strategy. It remains low during the initial period of certainty, spikes dramatically after the shock as beliefs transition from one confident state to another through a period of maximum confusion, and then decays as the new state is learned.

**Figure 3:**
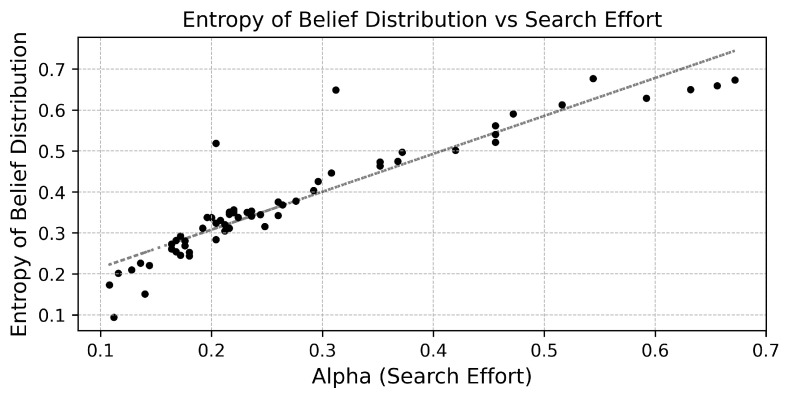
Information gathering effort as a function of subjective uncertainty. The figure plots the agent’s information gathering intensity ($\alpha_{t}$) against the corresponding entropy of their beliefs ($H_{t}$). The strong positive relationship confirms that effort allocated to learning is proportional to the agent’s uncertainty.

The direct relationship between uncertainty and information-seeking behavior is formalized in Figure 3, which plots the information-gathering intensity ($\alpha_{t}$) against the belief entropy ($H_{t}$) for each time period. The result is a strong, positive, and approximately linear relationship with Pearson correlation coefficient 0.928. This confirms the central hypothesis of our micro-foundational model; the agent endogenously allocates effort to learn about the environment in direct proportion to their uncertainty about it. This behavior is not a pre-programmed rule but an emergent consequence of the agent acting to minimize expected free energy. These results provide a micro-foundation for the link between a worker’s cognitive state and their observable search behavior.

## 4. A Macroeconomic Model of Labor Market Matching

Having established the micro-foundations of an individual’s search strategy, we now aggregate this behavior to analyze its macroeconomic implications. This section builds a macroeconomic model populated by a continuum of the active inference agents described previously. The central objective is to demonstrate how collective cognitive phenomena, specifically the distribution and accuracy of beliefs across the workforce, can act as an endogenous driver of aggregate labor market performance. We formalize this by linking the cognitive states of the unemployed population directly to the efficiency parameter of the aggregate matching function. In doing so, the model explains fluctuations in the job-finding rate and endogenizes a component that is typically exogenous in standard DMP models. The following analysis will define the composition of the unemployed pool, derive the aggregate matching technology, and specify the dynamic laws of motion for our key state variables.

### 4.1. Endogenizing Matching Efficiency

The aggregation of individual search behavior depends on how an agent’s cognitive state translates into effective job finding. To formalize this, we first structure the unemployed population. Let the total number of unemployed workers at time *t* be $U_{t}$. We partition this population into two distinct groups based on the alignment of their beliefs with the true state of the market, *S*. Let $U_{t}^{T}$ denote the measure of unemployed workers holding **T**rue beliefs that correspond to state *S*, and let $U_{t}^{F}$ be the measure of workers holding **F**alse beliefs. The total unemployed population is thus

(7) $$U_{t}=U_{t}^{T}+U_{t}^{F}.$$

From this, we define our primary cognitive state variable, $\lambda_{t}$, as the proportion of the unemployed workforce with misaligned beliefs:

(8) $$\lambda_{t}=\frac{U_{t}^{F}}{U_{t}}.$$

Let $\bar{f}\left(\theta_{t}\right)$ denote the baseline job finding rate per unit of effective search effort, where $\theta_{t}=v_{t}/u_{t}$ is labor market tightness. In the standard DMP model, it is typically defined as

(9) $$\bar{f}\left(\theta_{t}\right)=\bar{\mu }\theta_{t}^{1-\phi },$$

where $\bar{\mu }$ is the baseline matching efficiency and ϕ is the matching elasticity. The effective search effort of an agent *i* is the product of their application intensity (1−α) and an indicator I for their holding belief. The individual’s job-finding probability is therefore:

(10) $$p_{i,t}=\sum_{j\in \{T,F\}}\left(1-\alpha_{i}\right)\cdot I\left(\mathrm{type}(i)=j\right)\cdot {\bar{f}}^{j}\left(\theta_{t}\right).$$

To simplify this equation, we assume that almost only the search effort of correctly informed workers contributes to successful matches. The applications of workers with false beliefs are considered fundamentally misdirected and ineffective, yielding a very small match probability which can be ignored. This simplifying assumption represents a benchmark case where the gap between an incorrect model and market reality is sufficiently large that search efforts based on false premises are systematically unsuccessful, for instance, when workers search for jobs using obsolete skills in a technologically transformed industry.

Under this assumption, the total number of new matches in the economy, $M_{t}$, is the integral of these individual probabilities over the entire unemployed population with correct beliefs:

(11) $$M_{t}=\int_{i\in U_{t}^{T}}p_{i,t}di.$$

Let ${\bar{\alpha }}_{t}^{T}$ be the average information-gathering intensity among the group of workers with true beliefs. The aggregate matching function can then be expressed as

(12) $$M_{t}=U_{t}^{T}\cdot \left(1-{\bar{\alpha }}_{t}^{T}\right)\cdot \bar{f}\left(\theta_{t}\right).$$

The aggregate job-finding rate, $f_{t}=M_{t}/U_{t}$, is obtained by dividing the total matches by the total unemployed population. Substituting $U_{t}^{T}=\left(1-\lambda_{t}\right)U_{t}$, we arrive at our key result:

(13) $$f_{t}=\left(1-\lambda_{t}\right)\cdot \left(1-{\bar{\alpha }}_{t}^{T}\right)\cdot \bar{f}\left(\theta_{t}\right).$$

This expression formally endogenizes the efficiency of the labor market matching process. The matching efficiency is no longer a constant exogenous parameter, but is now explicitly a function of cognitive matching channel $C_{t}$, expressed by two endogenous cognitive variables, fluctuating with the collective beliefs and uncertainty of the workforce:

(14) $$\mu_{t}=\underset{\mathrm{Cognitive}\;\mathrm{matching}\;\mathrm{channel}\;C_{t}}{\underset{︸}{\left(1-\lambda_{t}\right)\cdot \left(1-{\bar{\alpha }}_{t}^{T}\right)}}\cdot \bar{\mu }.$$

The first, ($1-\lambda_{t}$), represents a cognitive composition effect: the share of the unemployed whose search is effective. The second, ($1-{\bar{\alpha }}_{t}^{T}$), represents a cognitive behavioral effect: the average search intensity of this effective group.

### 4.2. The Dynamics of Beliefs and Search Intensity

To analyze the model’s behavior away from a steady state, particularly in response to aggregate shocks, we must specify the laws of motion for the two endogenous cognitive variables that govern matching efficiency: the proportion of misinformed workers, $\lambda_{t}$, and the average information-gathering effort of the informed group, ${\bar{\alpha }}_{t}^{T}$. These dynamic equations are grounded in the principles of population dynamics and the micro-foundations of active inference established in Section 3.

#### 4.2.1. Law of Motion for $\lambda_{t}$

The evolution of $\lambda_{t}$, the share of the unemployed holding false beliefs, is determined by the flows into and out of the $U_{t}^{F}$ and $U_{t}^{T}$ states. Applying the time derivative to $\lambda_{t}$ yields

(15) $${\dot{\lambda }}_{t}=\frac{{\dot{U}}_{t}^{F}}{U_{t}}-\lambda_{t}\frac{{\dot{U}}_{t}}{U_{t}},$$

which can be expressed in terms of underlying population flows. We model these flows to include job separations, differential job-finding rates, and an active learning process. This yields a dynamic equation for $\lambda_{t}$ with three intuitive components (see Appendix C for the proof):

(16) $${\dot{\lambda }}_{t}=\underset{\mathrm{Inflow}\;\mathrm{Effect}}{\underset{︸}{\left(\lambda_{e}-\lambda_{t}\right)\cdot \frac{sE_{t}}{U_{t}}}}+\underset{\mathrm{Selection}\;\mathrm{Effect}}{\underset{︸}{\left(f_{T}-f_{F}\right)\cdot \lambda_{t}\left(1-\lambda_{t}\right)}}-\underset{\mathrm{Learning}\;\mathrm{Effect}}{\underset{︸}{k\cdot \lambda_{t}}}.$$

Each term has a distinct economic interpretation:

1. Inflow Effect: This term captures the change in $\lambda_{t}$ due to the inflow of newly unemployed workers from employment ($E_{t}$) at a separation rate *s*. If the fraction of new entrants with false beliefs, $\lambda_{e}$, differs from the current stock’s composition, $\lambda_{t}$, this inflow will pull the average composition towards $\lambda_{e}$.
2. Selection Effect: This term arises because workers with true beliefs ($U_{t}^{T}$) find jobs at a higher rate ($f_{T}$) than workers with false beliefs ($U_{t}^{F}$, with rate $f_{F}\approx 0$). This differential exit rate means that correctly informed workers leave the unemployment pool more quickly, which increases the concentration of misinformed workers among the remaining stock.
3. Learning Effect: This is the direct macroeconomic manifestation of the individual belief-updating process. It reflects that workers with false beliefs are not passive; they engage in information gathering, discover their model of the world is incorrect, and transition to the correctly informed state $U_{t}^{T}$. We model this as a flow out of $U_{t}^{F}$ occurring at a rate *k*, which represents the speed of cognitive adjustment or collective learning in the economy.

#### 4.2.2. Functional Form for Average Search Intensity ${\bar{\alpha }}_{t}^{T}$

The second dynamic component, ${\bar{\alpha }}_{t}^{T}$, is the average information-gathering effort of the correctly-informed group. While a full derivation would require integrating the complex policy function over the entire distribution of belief states, we can specify a reduced-form relationship that captures the primary insight from our micro-foundational model. The simulation in Section 3 demonstrated that an individual’s epistemic effort is proportional to their subjective uncertainty. We extend this principle to the aggregate level by positing that the average search intensity is a function of the overall uncertainty prevailing in the market.

We propose that a substitute for this aggregate uncertainty is the degree of belief dispersion within the unemployed population. A natural measure for this is the Shannon entropy of the distribution of workers between the “False” and “True” belief states, given by $\left[\lambda_{t},1-\lambda_{t}\right]$. This leads to the following functional form:

(17) $${\bar{\alpha }}_{t}^{T}=\alpha_{\mathrm{base}}+c\cdot H\left(\lambda_{t}\right),$$

where the Shannon entropy is

(18) $$H\left(\lambda_{t}\right)=-\left[\lambda_{t}\log_{2}\left(\lambda_{t}\right)+\left(1-\lambda_{t}\right)\log_{2}\left(1-\lambda_{t}\right)\right].$$

This specification decomposes the average search effort into two elements. The first, $\alpha_{\mathrm{base}}$, is a constant representing the baseline level of information gathering, which can be interpreted as a minimal monitoring cost that persists even in a stable and well-understood market. The second, time-varying component, $c\cdot H(\lambda_{t})$, directly reflects the core principle of active inference at the macro level. The parameter c>0 scales the sensitivity of search effort to aggregate uncertainty, which is captured by the entropy term $H(\lambda_{t})$. When the workforce is in broad agreement (either correctly, $\lambda_{t}\to 0$, or incorrectly, $\lambda_{t}\to 1$), entropy is low, and average information search is minimal. Conversely, when there is maximum disagreement and confusion about the state of the market ($\lambda_{t}\to 0.5$), entropy is at its peak, driving the highest level of collective epistemic search effort.

## 5. Analysis of the Macroeconomic Model

**Figure 4:**
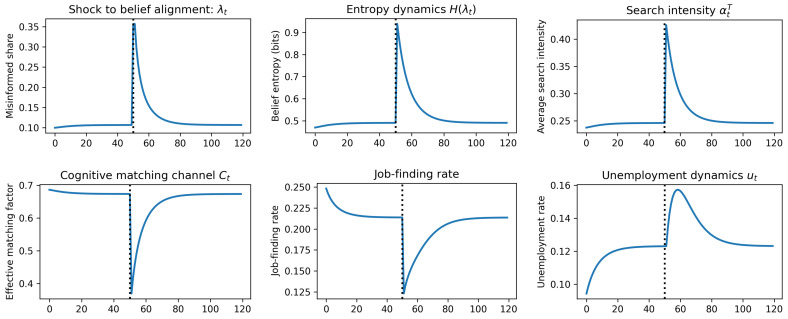
Illustration of system dynamics following an impulse shock to $\lambda_{t}$. The economy is initialized at its pre-shock steady state. At t=50 an unanticipated, one-off structural shock (indicated by the dashed lines) raises the misbelief share $\lambda_{t}$. We assume the vacancy rate is held constant. The panels report the ensuing impulse responses of belief misalignment, uncertainty (Shannon entropy), information effort, the cognitive matching channel, job finding, and unemployment.

**Figure 5:**
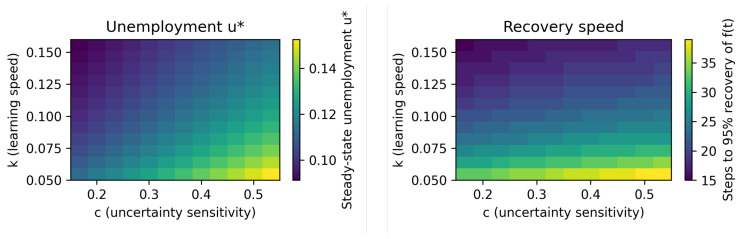
Heatmaps across (k,c) pair. Left heatmap reports the unemployment rate as a function of the learning speed *k* and the uncertainty sensitivity *c*; right heatmap shows the number of steps required for the job-finding rate to return to 95% of its pre-shock level.

With the model’s components fully specified, we now analyze its equilibrium properties and dynamic behavior. The framework’s primary contribution lies in its ability to generate endogenous labor market dynamics in response to structural economic changes. We first characterize the model’s long-run steady state, with a particular focus on the implications for matching efficiency and the Beveridge curve. We then analyze the system’s dynamic transition path following an aggregate structural shock, as illustrated in the impulse responses in Figure 4. Finally, we explore the policy implications and parameter sensitivities using comparative static heatmaps, shown in Figure 5.

In the absence of further shocks, the system converges to a steady-state equilibrium where the proportion of misinformed workers is constant (${\dot{\lambda }}_{t}=0$). This yields a steady-state value, $\lambda^{*}$, which is determined by the balance between the inflow of newly unemployed workers who may hold false beliefs, the more rapid exit of correctly informed workers from the unemployment pool, and the continuous process of active learning. The existence of a non-zero $\lambda^{*}$ implies that even in a stable economy, a degree of informational friction persists due to natural worker turnover and the inherent lags in cognitive adjustment. This long-run equilibrium is characterized by a constant level of average search intensity, ${\bar{\alpha }}_{t}^{T*}$, and a stable aggregate job-finding rate, reflecting a baseline level of mismatch inherent in the market.

The model’s most insightful dynamics are revealed when the economy is hit by an unexpected and permanent structural shock, such as a technological revolution or a sudden change in trade patterns. Such a shock fundamentally alters the “true” state of the market, instantly making the established beliefs of a significant portion of the workforce obsolete. This event translates into a discrete upward jump in the state variable $\lambda_{t}$, as a large measure of previously informed workers are now categorized as holding false beliefs. The immediate consequence, as dictated by the job-finding equation, is a sharp collapse in the matching efficiency of the labor market. This decline in the job-finding rate occurs instantaneously, even if the aggregate number of vacancies remains unchanged, as the search efforts of a large fraction of the workforce suddenly become ineffective.

Following the initial impact of the shock, the economy enters a complex dynamic adjustment phase. The sharp increase in $\lambda_{t}$ not only reduces the share of effective searchers but also seeds widespread market uncertainty. As $\lambda_{t}$ moves from a low value towards 0.5, the aggregate belief entropy, $H(\lambda_{t})$, increases significantly. This rise in collective uncertainty triggers an increase in the average information-gathering effort, ${\bar{\alpha }}_{t}^{T}$, as rational agents shift their focus from pragmatic applications to epistemic exploration. This behavioral response can create a secondary negative effect on matching, as the increase in learning effort ($1-{\bar{\alpha }}_{t}^{T}$) further suppresses the application intensity of the remaining effective searchers. The economy thus enters a period of collective re-learning, characterized by low matching rates and high information-seeking activity. Over time, as workers interact with the new environment, the active learning process (governed by the rate *k*) takes hold, and $\lambda_{t}$ begins its slow descent towards a new steady state. As beliefs gradually align with the new reality, entropy falls, reducing the need for intense epistemic search and allowing ${\bar{\alpha }}_{t}^{T}$ to return to its baseline. Figure 4 illustrates the response patterns of this system. This facilitates a slow, endogenous recovery of the aggregate matching efficiency.

These dynamics have significant implications for core macroeconomic phenomena, most notably the behavior of the Beveridge curve, which plots the relationship between unemployment and job vacancies. The sudden increase in belief dispersion ($\lambda_{t}$) causes a sharp drop in matching efficiency, which manifests as a large, immediate outward shift of the Beveridge curve. For any given level of vacancy postings, the economy now sustains a much higher rate of unemployment. The subsequent, slow recovery of matching efficiency, driven by the path-dependent process of collective learning, corresponds to a gradual and potentially hesitant inward shift of the curve. Our model thus provides a cognition-based mechanism that can account for the persistent unemployment and the slow recovery of matching efficiency that are often observed empirically for many years following major recessions and structural transformations. This suggests that the speed of labor market recovery may be constrained not only by aggregate demand but also by the slow and costly process of collective cognition.

Taken together, these mechanisms also suggest that policy should target the cognitive bottleneck that emerges after structural change. Two margins are central in our framework. First, accelerating belief alignment acts (higher *k*) directly on the learning flow that reduces the stock of misinformed searchers. Instruments may include timely guidance and counseling, modular re-skilling and certification with fast feedback, and coordination of job-search assistance with local demand signals. Second, improving the information environment (lower *c*) helps correctly informed workers waste less effort on epistemic activity, even when market-wide disagreement is high. This may be implemented by richer labor-market information, clearer vacancy–skill taxonomies, interoperable credentials, and platform designs that surface relevant matches. These measures operate on distinct margins and are potentially complementary; faster learning shortens the misalignment phase, while better information lowers the intensity of the learning detour itself. There are also trade-offs; interventions that raise learning may temporarily pull effort away from applications. Therefore, cost-effectiveness and targeting groups with higher misalignment are both important.

To illustrate these qualitative implications, Figure 5 provide two heatmaps as simple comparative statics under a fixed vacancy rate. The steady-state map shows that higher *k* and lower *c* are associated with lower u*. The recovery map (95% of pre-shock job-finding) indicates that both measures shorten the adjustment phase.

## 6. Discussion and Conclusions

This paper stands at the intersection of several key research areas: labor market search theory, the economics of information, and computational models of decision making. As established in Section 1, a central puzzle in the DMP tradition is that aggregate matching efficiency varies over time, often shifting dramatically following structural shocks [1,2]. Our primary contribution is to introduce a novel cognitive framework to provide alternative explanations for information frictions within a macroeconomic labor model.

To achieve this, we employed the Free Energy Principle (FEP) as our theoretical toolkit, as introduced in Section 2. The unique advantage of this framework is that it provides a computational theory of how agents select actions in a dynamic and uncertain world [9]. Crucially, it allows us to model the fundamental trade-off between exploration and exploitation in a principled way, without ad hoc assumptions about the cost of information or the utility of learning. This endogenously derived cognitive trade-off is the mechanism that regulates matching efficiency in our model.

The approach in this paper is philosophically aligned with the Rational Inattention (RI) literature [12,13,14]. RI models posit that agents face constraints on their ability to process information and optimally choose what to pay attention to, given that information is costly to acquire or process. This has been applied to various macroeconomic contexts, including business cycle models and price setting [15]. Our approach differs in a crucial aspect. RI models typically posit an exogenous cost function or a fixed capacity for information processing (e.g., a Shannon capacity channel). In contrast, the active inference framework does not rely on an explicit information cost. Instead, the drive for epistemic action—information gathering—emerges endogenously from the imperative to minimize prediction error (or surprise). Information has value not just instrumentally for a better pragmatic outcome, but intrinsically for reducing uncertainty about the agent’s model of the world. Our model therefore focuses on the epistemic value of information rather than its cost.

Our work also relates to broader themes in macroeconomics. The dynamics of our belief-updating variable, $\lambda_{t}$, connect to the vast literature on learning in macroeconomic models, which studies how agent learning can mitigate information frictions, shape economic dynamics, or convergence to rational expectations equilibrium [16,17]. Our framework contributes a cognition-based foundation for this learning dynamic. Furthermore, the concept that belief dispersion itself can be a crucial macroeconomic state variable echoes findings from the literature on global games and information coordination, where dispersed information can lead to strategic uncertainty and influence aggregate outcomes [18]. By synthesizing these disparate fields, this paper offers a novel perspective on how the cognitive processes of individual agents can aggregate to shape the macroeconomic landscape.

A broad set of facts supports our behavioral channel. First, online search and application data show that information-seeking and search intensity are measurable and cyclically sensitive. Google job-search indices improve unemployment forecasting and spike around labor market turns [19,20]; application-level data reveal systematic patterns in search intensity over the spell of unemployment [21]. Second, re-skilling and schooling are countercyclical; U.S. community college and higher-education enrollment rose notably during and after the Great Recession [22,23]. Third, active labor market policies that intensify information and learning—job, such as search assistance and training, tend to have positive medium-run effects and perform relatively well in recessions [24,25]. These patterns are consistent with our mechanism; after shocks that alter the structure of demand, workers temporarily divert effort from applications to belief updating (information acquisition, counseling, training), depressing effective matches until beliefs realign.

However, the framework presented here is a stylized representation of complex phenomena, and its limitations point toward several promising avenues for future research. Future research could relax the assumption of agent homogeneity by introducing heterogeneity in prior beliefs, learning capabilities, or risk preferences. What is more, our model is one-sided, focusing exclusively on the worker. A significant extension would be to develop a two-sided search model where firms are also active inference agents, learning about the quality and composition of the labor pool.

Finally, the theoretical predictions of this paper call for empirical and quantitative validation. Our future work on this topic will proceed along two specific streams of economic analysis: (1) an econometric analysis stream, designed to calibrate the model’s novel parameters and empirically test its predictions against labor market data; (2) a macroeconomic analysis stream, which will use the model for formal comparative statistics and business cycle analysis. Such research would further illuminate the crucial role of collective cognition in shaping the landscape of the modern labor market. 

## Data Availability

No new data were created or analyzed in this study. The simulation code supporting the conclusions of this article will be made available by the authors on request.

## Abbreviations

The following abbreviations are used in this manuscript:

DMP	Diamond–Mortensen–Pissarides Model
EFE	Expected Free Energy
FEP	Free Energy Principle
KL-divergence	Kullback–Leibler divergence
POMDP	Partially Observable Markov Decision Process
RI	Rational Inattention

## Appendix A. Mathematical Derivations of FEP

### Appendix A.1. Derivation of Variational Free Energy as an Upper Bound on Surprise

The goal of inference is to find an approximate posterior q(θ) that is close to the true posterior p(θ|s). The discrepancy is measured by the KL divergence:

(A1) $$D_{KL}\left[q\left(\theta \right)||p\left(\theta |s\right)\right]=\int q\left(\theta \right)\log\frac{q(\theta )}{p(\theta |s)}d\theta .$$

By definition, $D_{KL}\geq 0$. Expanding the logarithm:

(A2) $$D_{KL}\left[q\left(\theta \right)||p\left(\theta |s\right)\right]=E_{q(\theta )}\left[\log q\left(\theta \right)\right]-E_{q(\theta )}\left[\log p\left(\theta |s\right)\right].$$

Using Bayes’ rule,

(A3) $$p\left(\theta |s\right)=\frac{p(\theta ,s)}{p(s)},$$

we can write

(A4) logp(θ|s)=logp(s,θ)−logp(s).

Substituting this into Equation (A2):

(A5) $$D_{KL}\left[q\left(\theta \right)||p\left(\theta |s\right)\right]=E_{q(\theta )}\left[\log q\left(\theta \right)\right]-E_{q(\theta )}\left[\log p\left(s,\theta \right)-\log p\left(s\right)\right].$$

Since logp(s) does not depend on θ, the expectation $E_{q(\theta )}\left[\log p\left(s\right)\right]$ is simply logp(s).

(A6) $$D_{KL}\left[q\left(\theta \right)||p\left(\theta |s\right)\right]=\underset{F}{\underset{︸}{E_{q(\theta )}\left[\log q\left(\theta \right)-\log p\left(s,\theta \right)\right]}}+\log p\left(s\right).$$

Let us define variational free energy *F* as the first term. Rearranging the equation gives:

(A7) $$F=D_{KL}\left[q\left(\theta \right)||p\left(\theta |s\right)\right]-\log p\left(s\right).$$

Since $D_{KL}\geq 0$, it follows directly that F≥−logp(s). Minimizing *F* thus minimizes the KL divergence between the approximate and true posterior, making *F* a tight upper bound on surprise.

### Appendix A.2. Decomposition of Free Energy (F)

The decomposition of *F* into complexity and accuracy follows from expanding the joint probability p(s,θ)=p(s|θ)p(θ) within its definition.

(A8) $$\begin{array}{c} \begin{array}{cc} F & =E_{q(\theta )}\left[\log q\left(\theta \right)-\log p\left(s,\theta \right)\right]\\ \; & =E_{q(\theta )}\left[\log q\left(\theta \right)-\log p\left(s|\theta \right)-\log p\left(\theta \right)\right]\\ \; & =E_{q(\theta )}\left[\log q\left(\theta \right)-\log p\left(\theta \right)\right]-E_{q(\theta )}\left[\log p\left(s|\theta \right)\right]\\ \; & =\underset{\mathrm{Complexity}}{\underset{︸}{D_{KL}\left[q(\theta )||p(\theta )\right]}}-\underset{\mathrm{Accuracy}}{\underset{︸}{E_{q(\theta )}\left[\log p(s|\theta )\right]}}. \end{array} \end{array}$$

### Appendix A.3. Decomposition of Expected Free Energy (G)

The expected free energy, *G*, associated with an action, $a_{t}$, is formally the free energy that is expected to arise in the future, given that action. This involves an expectation over all possible future outcomes ($o_{t+1}$) and the hidden states ($s_{t+1}$) that might cause them. We begin with the definition of future free energy, which is the free energy associated with a future observation $o_{t+1}$:

(A9) $$F\left(o_{t+1}\right)=E_{q(s_{t+1}|o_{t+1})}\left[\log q\left(s_{t+1}|o_{t+1}\right)-\log p\left(o_{t+1},s_{t+1}\right)\right]$$

The expected free energy $G(a_{t})$ is the expectation of this quantity over the outcomes that the agent predicts will follow action $a_{t}$:

(A10) $$\begin{array}{c} \begin{array}{cc} G(a_{t}) & =E_{q(o_{t+1}|a_{t})}\left[F\left(o_{t+1}\right)\right]\\ \; & =E_{q(o_{t+1}|a_{t})}\left[E_{q(s_{t+1}|o_{t+1})}\left[\log q\left(s_{t+1}|o_{t+1}\right)-\log p\left(o_{t+1},s_{t+1}\right)\right]\right] \end{array} \end{array}$$

Using the law of iterated expectations, we can write this as a single expectation over the joint distribution:

(A11) $$G\left(a_{t}\right)=E_{q(s_{t+1},o_{t+1}|a_{t})}\left[\log q\left(s_{t+1}|o_{t+1}\right)-\log p\left(o_{t+1},s_{t+1}\right)\right]$$

To reveal the pragmatic and epistemic components, we perform a key decomposition on the term from the generative model, $p(o_{t+1},s_{t+1})$. We use the chain rule of probability,

(A12) $$p\left(o_{t+1},s_{t+1}\right)=p\left(o_{t+1}|s_{t+1}\right)p\left(s_{t+1}\right).$$

Substituting this into the equation for $G(a_{t})$:

(A13) $$G\left(a_{t}\right)=E_{q(s_{t+1},o_{t+1}|a_{t})}\left[\log q\left(s_{t+1}|o_{t+1}\right)-\log p\left(s_{t+1}\right)-\log p\left(o_{t+1}|s_{t+1}\right)\right]$$

Adding and subtracting the prior beliefs $\log q(s_{t+1}|a_{t})$:

(A14) $$\begin{array}{c} \begin{array}{cc} G(a_{t})= & E_{q(s_{t+1},o_{t+1}|a_{t})}\left[\log q\left(s_{t+1}|o_{t+1}\right)-\log q\left(s_{t+1}|a_{t}\right)\right]\\ \; & +E_{q(s_{t+1}|a_{t})}\left[\log q\left(s_{t+1}|a_{t}\right)-\log p\left(s_{t+1}\right)\right]\\ \; & +E_{q(s_{t+1},o_{t+1}|a_{t})}\left[-\log p\left(o_{t+1}|s_{t+1}\right)\right] \end{array} \end{array}$$

Here, the middle term is the KL divergence between $q(s_{t+1}|a_{t})$ and $p(s_{t+1})$. Assuming $p\left(s_{t+1}\right)\approx q\left(s_{t+1}|a_{t}\right)$, the KL divergence term becomes approximately zero. Recognizing that preferences are encoded as priors over observations $p(o_{t+1})$, finally we get:

(A15) $$G\left(a_{t}\right)=\underset{\mathrm{Pragmatic}\;\mathrm{Value}}{\underset{︸}{E_{q(o_{t+1}|a_{t})}\left[-\log p\left(o_{t+1}\right)\right]}}+\underset{\mathrm{Epistemic}\;\mathrm{Value}}{\underset{︸}{E_{q(o_{t+1},s_{t+1}|a_{t})}\left[\log q\left(s_{t+1}|o_{t+1}\right)-\log q\left(s_{t+1}|a_{t}\right)\right]}}.$$

## Appendix B. The Microeconomic Simulation

### Appendix B.1. Model Framework

We model the worker as an agent operating within a Partially Observable Markov Decision Process (POMDP). The agent’s goal is not to maximize a reward function, but to minimize its long-term Expected Free Energy (EFE) by actively making inferences about the hidden state of its environment.

The model is defined by the following components:

- State Space (S): A finite set of unobservable (hidden) states of the world.
- Action Space (A): A finite set of actions available to the agent.
- Observation Space (O): A finite set of observations the agent can receive from the environment.
- Generative Model (*p*): The agent’s internal, probabilistic model of how the world works, which is a joint probability distribution over states, actions, and observations, $P(s_{t+1},s_{t},o_{t},a_{t})$. This is composed of specific conditional probabilities described below.

### Appendix B.2. Generative Model Specification

The agent’s “worldview” is formally specified by the following components:

#### State Space (S):

The hidden state of the world, $s_{t}$, represents the true state of the labor market at time *t*. We define two possible states:

(A16) $$S=\left\{s^{\left(A\right)},s^{\left(B\right)}\right\},$$

where $s^{\left(A\right)}$ represents the “Old Economy” where Skill A is in demand, and $s^{\left(B\right)}$ represents the “New Economy” where Skill B is in demand.

#### Action Space (A):

At each time step, the agent chooses an action $a_{t}\in A$ that corresponds to allocating its effort between job application and information search. We define a discrete set of policies based on the search intensity parameter α:

(A17) $$A=\left\{\alpha_{L},\alpha_{M},\alpha_{H}\right\}=\left\{0.1,0.5,0.9\right\},$$

where $\alpha_{L}$ represents a strong focus on job application, and $\alpha_{H}$ represents a strong focus on information search.

#### Observation Space (O):

The agent receives observations $o_{t}\in O$ that provide clues about the hidden state. The observation space is defined as:

(A18) $$O=\left\{\mathrm{no}\;\mathrm{offer},\mathrm{low}\;\mathrm{offer}\;A,\mathrm{high}\;\mathrm{offer}\;A,\mathrm{low}\;\mathrm{offer}\;B,\mathrm{high}\;\mathrm{offer}\;B,\mathrm{news}\;A,\mathrm{news}\;B\right\},$$

#### Probabilistic Mappings:

The agent’s model is parameterized by the following probability distributions:

- Likelihood Mapping (A-matrix): This defines the agent’s belief about the probability of an observation given the current state and the action taken. This is represented by a tensor A of dimensions |O|×|S|×|A|, where the elements are $p(o_{t}|s_{t},a_{t})$. Crucially, the agent’s model associates specific actions with specific outcomes. For example, for a “pragmatic” action ($a_{t}=\alpha_{L}$), the agent believes:
  − $p(o_{t}=“\mathrm{high}\;\mathrm{offer}\;A”|s_{t}=s^{\left(A\right)},a_{t}=\alpha_{L})$ is high.
  − $p(o_{t}=“\mathrm{no}\;\mathrm{offer}”|s_{t}=s^{\left(B\right)},a_{t}=\alpha_{L})$ is very high. This represents the agent’s “smart” understanding that applying for A-type jobs in a B-type economy will likely result in failure. For an “epistemic” action ($a_{t}=\alpha_{H}$), the agent believes observations will be of type “news” with the content of the news depending on the true state, e.g., $p(o_{t}=“\mathrm{news}\;B”|s_{t}=s^{\left(B\right)},a_{t}=\alpha_{H})$ is high.
- State Transitions (B-matrix): This defines the agent’s belief about the probability of the market transitioning between states, $p(s_{t+1}|s_{t})$. This is represented by a matrix B of dimensions |S|×|S|. We model the agent as being aware of market volatility, such that:
  (A19) $$p\left(s_{t+1}=j|s_{t}=i\right)=B_{ij}=\left\{\begin{array}{cc} 1-\epsilon & \;\mathrm{if}\;j=i\\ \epsilon & \;\mathrm{if}\;j\neq i \end{array}\right.$$
  where ϵ is a small constant representing the perceived probability of a structural shock.
- Prior Preferences (C-vector): These encode the agent’s goals as a categorical distribution over desired observations, p(o). We denote this by a vector C of length |O|. Observations like “high offer A” and “high offer B” are assigned high preference values, while “no offer” is assigned a low value.

### Appendix B.3. Inference and Decision Making

#### Perceptual Inference (Belief Update)

The agent maintains a probabilistic belief over the hidden states, $q(s_{t})$, which is an approximation of the true posterior $P(s_{t}|o_{1:t})$. Upon receiving a new observation $o_{t}$ after taking action $a_{t-1}$, the agent updates its belief via a simplified form of Bayesian filtering:

1. Prediction: The prior belief for the current state is formed based on the previous state and the transition model:
  (A20) $$q\left(s_{t}|q\left(s_{t-1}\right)\right)=\sum_{s_{t-1}}P\left(s_{t}|s_{t-1}\right)q\left(s_{t-1}\right)=Bq\left(s_{t-1}\right)$$
2. Update: The predicted belief is updated using the likelihood of the new observation:
  (A21) $$q\left(s_{t}\right)\propto P\left(o_{t}|s_{t},a_{t-1}\right)\cdot q\left(s_{t}|q\left(s_{t-1}\right)\right)$$

#### Active Inference (Action Selection)

The agent selects the action $a_{t}$ that is expected to minimize the free energy of future outcomes. For a one-step-ahead agent, this corresponds to choosing the action that minimizes the Expected Free Energy ($G(a_{t})$):

(A22) $$a_{t}^{*}=\arg\min_{a_{t}}G\left(a_{t}\right).$$

For this simulation, the agent’s policy, $\pi (a_{t})$, uses a softmax function to select actions stochastically based on their EFE,

(A23) $$\pi \left(a_{t}\right)=\frac{e^{-\beta \cdot G(a_{t})}}{\sum_{a^{\prime }\in A}e^{-\beta \cdot G(a^{\prime })}},$$

where β is a precision parameter controlling the level of determinism.

### Appendix B.4. Simulation Experiment

The simulation is designed to test the agent’s adaptability to an unannounced structural shock.

- Initial Conditions (t=1 to 40): The true market state is $s^{\left(A\right)}$ (“Old Economy”). The agent begins with a strong prior belief $q\left(s_{0}=s^{\left(A\right)}\right)\approx 1$.
- Shock (t=41): The true market state exogenously and permanently switches to $s^{\left(B\right)}$ (“New Economy”).
- Adaptation Phase (t=41 to 100): We observe the agent’s behavior—specifically its beliefs $q(s_{t})$ and its chosen search intensity $\alpha_{t}t$—as it encounters surprising evidence and adapts to the new reality.

## Appendix C. Mathematical Derivations of ${\dot{\lambda }}_{t}$

We start from the time derivative of $\lambda_{t}$. According to Equation (15),

(A24) $${\dot{\lambda }}_{t}=\frac{{\dot{U}}_{t}^{F}}{U_{t}}-\lambda_{t}\frac{{\dot{U}}_{t}}{U_{t}}.$$

The derivation proceeds by defining the expressions for the net flows ${\dot{U}}_{t}^{F}$ and ${\dot{U}}_{t}$.

### Appendix C.1. Law of Motion for the Number of F-Believers ${\dot{U}}_{t}^{F}$

The net change in the stock of unemployed F-believers is the sum of inflows minus outflows.

- Inflows: The only inflow consists of newly unemployed workers who held false beliefs while employed.
  (A25) $$\mathrm{Inflow}=s\cdot E_{t}^{F}=s\cdot \lambda_{e}E_{t}.$$
- Outflows: There are two outflows for this group.
  − Finding a job: Workers in this group find jobs at a rate $f_{F}$.
    (A26) $${\mathrm{Outflow}}_{1}=f_{F}\cdot U_{t}^{F}.$$
  − Cognitive transition: Workers in this group revise their beliefs from F to T due to persistent surprise. This occurs at a rate *k* when a mismatch exists.
    (A27) $${\mathrm{Outflow}}_{1}=k\cdot U_{t}^{F}.$$

Combining these flows yields the net change in $U_{t}^{F}$:

(A28) $${\dot{U}}_{t}^{F}=s\cdot \lambda_{e}E_{t}-f_{F}\cdot U_{t}^{F}-k\cdot U_{t}^{F}.$$

### Appendix C.2. Law of Motion for the Total Unemployed Population ${\dot{U}}_{t}$

Similarly, the net change in the total unemployed population is the total inflow of newly unemployed workers minus the total outflow of all workers who find jobs.

- Total Inflows:
  (A29) $$\mathrm{Inflow}=s\cdot E_{t}$$
- Total Outflows: The total number of matches, $M_{t}$, is given by $M_{t}=f_{t}U_{t}$.
  (A30) $$\mathrm{Outflow}=f_{t}U_{t}=\left(\lambda_{t}f_{F}+\left(1-\lambda_{t}\right)f_{T}\right)\cdot U_{t}$$

Combining these yields the net change in $U_{t}$:

(A31) $${\dot{U}}_{t}=s\cdot E_{t}-\left(\lambda_{t}f_{F}+\left(1-\lambda_{t}\right)f_{T}\right)\cdot U_{t}.$$

### Appendix C.3. Substitution and Simplification

We now substitute Equations (A28) and (A31) into our starting Equation (A24).

(A32) $${\dot{\lambda }}_{t}=\frac{s\cdot \lambda_{e}E_{t}-f_{F}\cdot U_{t}^{F}-k\cdot U_{t}^{F}}{U_{t}}-\lambda_{t}\frac{s\cdot E_{t}-\left(\lambda_{t}f_{F}+\left(1-\lambda_{t}\right)f_{T}\right)\cdot U_{t}}{U_{t}}.$$

Substitute $U_{t}^{F}=\lambda_{t}U_{t}$ and separate the terms:

(A33) $$\begin{array}{c} \begin{array}{cc} {\dot{\lambda }}_{t} & =\frac{s\cdot \lambda_{e}E_{t}}{U_{t}}-\frac{f_{F}\cdot \lambda_{t}U_{t}}{U_{t}}-\frac{k\cdot \lambda_{t}U_{t}}{U_{t}}-\left(\frac{s\cdot \lambda_{t}E_{t}}{U_{t}}-\frac{\lambda_{t}\cdot \left(\lambda_{t}f_{F}+\left(1-\lambda_{t}\right)f_{T}\right)\cdot U_{t}}{U_{t}}\right)\\ \; & =\frac{s\cdot \lambda_{e}E_{t}}{U_{t}}-f_{F}\cdot \lambda_{t}-k\cdot \lambda_{t}-\frac{s\cdot \lambda_{t}E_{t}}{U_{t}}+\lambda_{t}\cdot \left(\lambda_{t}f_{F}+\left(1-\lambda_{t}\right)f_{T}\right) \end{array} \end{array}$$

Next, we group similar terms. First, group the terms related to job separation:

(A34) $$\frac{s\cdot \lambda_{e}E_{t}}{U_{t}}-\frac{s\cdot \lambda_{t}E_{t}}{U_{t}}=\left(\lambda_{e}-\lambda_{t}\right)\frac{sE_{t}}{U_{t}}$$

Then group the terms related to job-finding rates:

(A35) $$\begin{array}{c} \begin{array}{cc} -f_{F}\cdot \lambda_{t}+\lambda_{t}\cdot \left(\lambda_{t}f_{F}+\left(1-\lambda_{t}\right)f_{T}\right)\; & =-\lambda_{t}f_{F}+\lambda_{t}^{2}f_{F}+\lambda_{t}\left(1-\lambda_{t}\right)f_{T}\\ \; & =-\lambda_{t}f_{F}\left(1-\lambda_{t}\right)+\lambda_{t}\left(1-\lambda_{t}\right)f_{T}\\ \; & =\lambda_{t}\left(1-\lambda_{t}\right)\left(f_{T}-f_{F}\right) \end{array} \end{array}$$

Substituting this back into the main expression (A33) gives us the final differential equation:

(A36) $${\dot{\lambda }}_{t}=\underset{\mathrm{Inflow}\;\mathrm{Effect}}{\underset{︸}{\left(\lambda_{e}-\lambda_{t}\right)\cdot \frac{sE_{t}}{U_{t}}}}+\underset{\mathrm{Selection}\;\mathrm{Effect}}{\underset{︸}{\left(f_{T}-f_{F}\right)\cdot \lambda_{t}\left(1-\lambda_{t}\right)}}-\underset{\mathrm{Learning}\;\mathrm{Effect}}{\underset{︸}{k\cdot \lambda_{t}}}.$$
